# Influence of Genotype on Productivity and Egg Quality of Three Hen Strains Included in a Biodiversity Program

**DOI:** 10.3390/ani13111848

**Published:** 2023-06-01

**Authors:** Jolanta Calik, Joanna Obrzut

**Affiliations:** Department of Poultry Breeding, National Research Institute of Animal Production, Krakowska Street 1, 32-083 Krakow, Poland; joanna.obrzut@iz.edu.pl

**Keywords:** hens, biodiversity, health, productivity, egg quality

## Abstract

**Simple Summary:**

As a result of the increasing intensification and globalization of poultry production, the number of poultry breeds/varieties is consistently declining. The elimination of populations well adapted to local conditions risks losing desirable traits such as high survival rates, resistance to diseases and adverse environmental conditions, and high reproductive capacities. Production based on local breeds can be useful for use in agriculturally poor regions, contributing to the effective management of these areas and to the production of eggs and meat with a unique nutrition and taste. An analysis of the performance and egg quality of three hen strains included in a conservation program in Poland was carried out. It was shown that the random mating system used in the reproduction of flocks effectively protects populations from an increase in the degree of inbreeding. Hens kept in small, closed populations, in which selection aimed at improving performance traits was not carried out, retained their genetic distinctness, manifesting, among other things, in significant differences in performance traits and egg quality.

**Abstract:**

The aim of the study was to evaluate the effect of genotype on the productivity and egg quality of three hen strains included in the genetic resource protection program in Poland. The study encompassed populations of laying hens, i.e., Rhode Island Red/RIR (R-11 and K-22) and Rhode Island White (A-33). The analysis over five generations included the basic production traits, i.e., the weight of the birds at 20 weeks (g), egg weights at 33 and 53 weeks, sexual maturity, the number of eggs laid up to 56 weeks of age, and hatchability parameters. In addition, the effective population size (N_e_) and flock homozygosity coefficient (F_x_) were calculated for each breed. Population health during the rearing and production periods was also recorded. The study also determined egg content and shell quality traits in relation to the age of the hens. The birds were kept on litter at a stocking rate of 5 hens/m^2^ and fed ad libitum with a standard feed mixture for hens. Based on the results, it was concluded that the evaluated hen populations (R-11, K-22, and A-33) are valuable strains, representing a reservoir of unique phenotypic and egg quality traits. It was shown that the random mating system used in the reproduction of flocks effectively protects the populations from an increase in the degree of inbreeding. An influence of hens’ origin (genotype) and age on the performance results, as well as egg quality traits, was found. Over five generations, the evaluated hen strains were characterized by high survival rates (above 98%). The study also found a large variation between the R-11 and K-22 strains and the A-33 strain in terms of the evaluated performance traits, especially in the body and egg weights, sexual maturity age, and laying performance of hens. The earliest to start laying were hens from the K-22 strains, which also showed significantly (*p* ≤ 0.05) higher laying performances compared to R-11 hens. The results also indicate that the quality of eggs from hens of the compared strains varied. This was particularly true for such traits as shell color; egg, shell, and yolk weight; and shell quality. It was also shown that many egg and shell quality traits change with the age of the hens. The analysis of the obtained data indicates that the adopted methods of conservative breeding for these populations have influenced the success of the conservation program.

## 1. Introduction

Livestock breeding has led to a significant reduction in genetic variability [1]. Industrial methods of livestock production have resulted in global production relying on a small number of high-yielding breeds, and traditional farming systems with native breeds are being marginalized [2,3,4]. According to the Food and Agriculture Organization (FAO), in Europe and the Caucasus, 53% of native breeds of farmed and domesticated animals are threatened by extinction [5]. The situation is particularly unfavorable in poultry production, where the often uncontrolled pursuit of maximum production and increasing profitability, e.g., by obtaining the highest number of eggs or high weight gains with the lowest possible feed consumption, has led to the extinction or significant depletion of many native breeds/strains of hens [2,4,6,7]. Meanwhile, the preservation of native poultry breeds is of great importance both in highly developed countries, where it is part of the policy recommendations for sustainable agricultural development, and in underdeveloped countries, where the rearing of native poultry dominates [1,8]. Hence, in recent years, we have seen an increasing number of studies on the characteristics of native and locally adapted breeds of hens from different parts of the world [9,10,11,12,13,14,15,16]. The authors especially point out the necessity of monitoring the effective population size and the inbreeding level of small, closed populations of hens, and emphasize that the maintenance of these parameters at an appropriate level determines their good health and productivity and allows assessing the effectiveness of adopted conservation programs.

In Poland, a number of native and locally adapted hen breeds have already become extinct, including, among others, gray hens, spotted hens, naked neck hens, and some varieties of crested hens. Hence, in the 1970s, the staff of the National Research Institute of Animal Production, with the participation of other scientific centers, defined the concept of conservation and genetic reserve flocks and developed the first conservation programs for the preservation of native breeds of hens, which included, among others, methods of conservation, breed standards, and an appropriate system for mating birds [2,6,7,16]. In the 1990s, on the initiative of the FAO, a comprehensive program for the conservation of genetic resources in agriculture was developed, the main goal of which was to preserve livestock populations threatened with extinction. It was signed by Poland along with 190 countries around the world. At that time, it was also decided to create the Global Databank for Farm Animal Genetic Resources and the Domestic Animal Diversity Information System (DAD-IS), and based on the information contained in this Global Databank, a catalog of genetic resources, known as the World Watch List for Domestic Animal Diversity, was developed [17].

Currently, Poland has a valuable collection of breeds/strains of hens (eight breeds/thirteen strains) included in the “Genetic resources conservation program for laying hen populations”, most of which were included by the FAO in the global genetic resources subject to conservation [17]. As a result of observations and studies, as well as adopted global solutions, and based on data from the implementation of conservation programs at the National Research Institute of Animal Production, a model for estimating the risk status of native breeds was developed and adapted to Polish conditions. Based on the number of males and females entered in the books and the value of the risk status indicator in all the above-mentioned hen populations, it was concluded that these breeds/strains currently require further protection [18].

These populations are perfectly adapted to harsh environmental conditions and provide excellent material for physiological studies of vital mechanisms, as well as for studies of the evolutionary history of farm animals based on work in immunogenetics, cytogenetics, serum protein polymorphism, and molecular genetics methods [19,20,21]. They are morphologically and productionally diverse, provide products, i.e., meat [22,23,24] and eggs [25,26,27,28], with unique dietary and taste value, and are invaluable genetic resources that can be used in future genetic improvement programs for commercial chicken flocks [2,6]. Some of the protected breeds of hens were imported to Poland more than forty years ago, and hens locally adapted to our production conditions are completely different from their present-day counterparts in their countries of origin [7,25]. Among them, a valuable group of birds is the Rhode Island Red breed, which is one of the most typical representatives of general purpose breeds, formerly widely distributed in Poland and known as the Crimson. The breed was developed in the second half of the 19th century in the state of Rhode Island in the United States as a result of crossbreeding various breeds of hens with Asian birds, such as Cochins and Malays, and conducting selection for a higher laying capacity. The R-11 strain was imported to Poland from Great Britain before 1939, while breeding work on the Rhode Island Red (K-22) and Rhode Island White (A-33) breeds began in Poland in the 1970s [26]. R-11 and K-22 hens and roosters have reddish-brown or mahogany plumage, while A-33 have white plumage and are not very fearful birds with a mild temperament. These birds are distinguished by their different genetic structure and ancestry compared to other strains kept in Poland and show a high degree of heterosis when crossed with other strains. These populations are particularly suitable for extensive, backyard farming, making excellent use of green paddocks. This is of particular importance, because in recent years there has been growing consumer interest in purchasing poultry products obtained from native breeds kept in extensive rearing systems [6,7].

Hen eggs are one of the most valuable products of animal origin, valued for their nutritional qualities, i.e., high biological contents of protein; vitamins, especially fat soluble vitamins A, D, E, and K; and valuable minerals such as calcium, manganese, iron, and zinc, and their wide application, mainly in the food industry, but also in the pharmaceutical and cosmetic industries [27,28]. The physical characteristics and chemical composition of eggs are influenced, among other things, by the origin of the laying hens, which, in addition to nutrition, age, or housing system, is a key determinant of the nutritional value of eggs [29,30,31,32,33,34,35,36,37]. Consumers increasingly pay attention not only to the price of eggs, but also to their taste, freshness, yolk and shell color, nutritional value, and health-promoting qualities, including, among others, their low cholesterol and increased content of unsaturated fatty acids and vitamins [27,34,37,38]. The most important characteristics in the commercial market are the weight of the egg and shell quality parameters, including the weight, thickness, and density of the shell, which affect its strength [28,39,40,41].

Production traits such as body weight, egg weight, and laying rate in hen populations included in the biodiversity program show high variability between genetically different populations of the same species [6,15,16,25,26], so it is very important to analyze their formation over several generations. The analysis of genetic and production parameters of three laying hen strains included in the biodiversity program is in line with global research in this area and can be a valuable source of information for the scientific community dealing with animal biodiversity. Such studies make it possible to assess the effectiveness of the adopted conservation program, and the results can be used to develop long-term strategies for the conservation of native breeds. The characteristics of egg quality regarding consumption are well understood in flocks of high-producing hens, while it is interesting to analyze the characteristics of egg quality parameters in flocks where no selection is carried out and flocks have been maintained in small populations for many generations.

The aim of this study was to evaluate the influence of genotype on the productivity and egg quality of three hen strains included in the genetic resource conservation program in Poland.

## 2. Materials and Methods

The study was carried out in 2018–2022 (5 generations) and included 3 populations of hens covered by the genetic resource conservation program, i.e., Rhode Island Red strains R-11 and K-22 and Rhode Island White strain A-33, maintained at the Experimental Station of the National Research Institute of Animal Production in Chorzelów, Poland. The study covered both the rearing period and the laying production period. The evaluation carried out was not an experimental procedure and therefore did not require applications and approval from the Ethical Committee.

Populations of hens over 5 generations during the production period were maintained in 4 groups/replicates in the following numbers:

- R-11: 804–900 birds including 84–100 roosters (21–25 each) and 720–800 hens (180–200 each);
- K-22: 1056 birds including 108 roosters (27 each) and 948 hens (237 each);
- A-33: 960 birds including 96 roosters (24 each) and 864 hens (216 each).

Throughout the rearing and production period, roosters and hens were maintained under optimal environmental conditions in accordance with zootechnical and welfare standards:

- Temperature: 32 °C during the first days of rearing, gradually decreasing as birds age to reach 16–20 °C after 35 days of age;
- Relative humidity: 65–70%;
- Lighting regime: 10–15 l×.
- Housing system: litter, stocking density of 5 birds/m^2^, and an enriched environment (perches, additional bales of straw, scratching areas, sand baths, claw-trimming areas, and grit).

Birds were fed ad libitum with standard complete feed mixtures and also had free access to water. The feed mixture contained:

1 day to 8 weeks: 18.04% total protein, 5.70% crude ash, 3.09% crude fat, 3.56 crude fiber, 0.90% calcium, and 0.63% phosphorus;
9–20 weeks: 17.05% total protein, 5.09% crude ash, 3.00% crude fat, 3.95 crude fiber, 0.76% calcium, and 0.57% phosphorus;
21–56 weeks: 16.93% total protein, 11.28% crude ash, 2.15% crude fat, 2.50% crude fiber, 3.55% calcium, and 0.50% phosphorus.

In each strain and generation, the effective population size (N_e_)—which depends on the number of females and males in the flock, depicting the rate of gene elimination due to random genetic drift—was estimated based on Wright’s formulas and the increase in flock homozygosity (F_x_), which is inversely proportional to the effective population size [42]. On the basis of breeding records (databases) and measurements taken, the following traits were analyzed: health of birds during the rearing and production period, body weight of birds at 20 weeks of age (hens n_group_ = 72−95 and roosters n_group_ = 28−36), and egg weight at 33 and 53 weeks of age (n_group_ =28) in every generation. The sexual maturity of the flocks was determined by the number of days of age of the hens counted from the day they were hatched until the flock reached 30% egg production, the number of eggs laid during the production period, i.e., 56 weeks of age (36 weeks of production), and the hatchability parameters (fertilization of eggs and hatchability of healthy chicks from set and fertilized eggs) as an average of five generations. Hatching was carried out in an incubator (Petersime, Belgium), following all recommendations for both temperature and relative humidity, which were as follows:

- Setting compartment, i.e., days 1–18 of incubation: 37.6–38.0 °C and 50–60% relative humidity, with the eggs rotated every hour through an angle of 90°.
- Hatching compartment, i.e., days 19–21 of incubation: 37.0–37.5 °C and 50–60% relative humidity after egg transfer and 75–80% during hatching of chicks.

From each population, 32 eggs laid on a single day (from 4 groups/replicates of 8 eggs each) were collected and subjected to a quality assessment using an electronic EQM (Egg Quality Measurement) apparatus (Technical Services and Supplies, York, England). Egg quality analyses were performed twice, i.e., at 33 and 53 weeks of the hens’ age (a total of 96 eggs in each test). The following parameters were included in the evaluation: egg, yolk, and shell weights (Ohaus scale with 0.01 g accuracy); shell color (QCR Shell Colour Reflectometer, percentage reading between black and white with the former expressed as 0% and the latter as pure white 100%); thick albumen height (measured in mm with an EQM detector after an egg was broken onto a mirrored table by contact of the EQM sensor with the surface of the dense albumen; accuracy of 0.1 mm); automatically calculated Haugh units (HU) (QCM), Roche yolk color score (QCC Colorimeter, using colorimeter according to 15-point scale, YolkFanTM, DSM Nutritional Products, Basel, Switzerland); shell thickness (μm) was (measured after removing shell membranes using a Mitutoyo Digital Micrometer 395–371 (Mitutoyo, Japan)), and shell density (calculated automatically from egg weight and shell thickness, mg/cm^2^). Blood and meat spots (%) in egg whites and yolks were recorded on a mirrored table as absent or present (0–1). The egg shape index was expressed as the ratio of the length (mm) of the short axis to the long axis measured with an electronic caliper (%), while the shell strength in a vertical position (N) was measured using a TA.XT Plus apparatus (Stable Micro Systems, Godalming, Surrey, UK) fitted with a 30 kg load cell and a 77 mm compression plate (*p*/75). The eggs were compressed at a constant test speed of 2 mm/min and the breaking strength was determined at the time of eggshell fracture.

For the body weight of birds at 20 weeks and the egg weight at 33 and 53 weeks, using linear regression equations and taking into account the R^2^ coefficient, the significance of the trend line (*p*), standard error of estimation (Se), and time trends were determined according to the formula:

y=ax+b

where y—the level of the trait (dependent variable);

a—linear regression coefficient;
x—time expressed in years (independent variable);
b—the level of the trait in the zero period.

The data are presented graphically (Excel), while the estimation of the statistical parameters of the regression model for the determination of the trend line was performed using the Statistica 13.3 statistical package [43]. The sexual maturity of the flock, the number of eggs laid during the production period, and the hatchability parameters are presented as an average of five generations. The mean values for all analyzed parameters were calculated by a one-way ANOVA. Statistical significance was considered to be *p* ≤ 0.05. Egg quality parameters were analyzed using Statistica 13.3 PL software. The mean values for all analyzed parameters were calculated. A two-way ANOVA was used to analyze variability (variable 1: genotype and variable 2: week). The significance of differences was verified using Duncan’s test. Interactions between experimental variables were assessed. Statistical significance was considered to be *p* ≤ 0.05.

## 3. Results

As can be seen from Table 1, the average effective size (N_e_) of the evaluated populations, which depends on the number of males and females, ranged from 311.83 to 387.82, which had a direct impact on the low level of flock inbreeding (F_x_), the average value of which ranged from 0.13 (K-22) to 0.16 (R-11). During the rearing period, the mortality as well as the health-related culling of the evaluated populations over five generations were low and did not exceed 1.20% for males and 2.10% for females. Additionally, during the production period, the survival rate of roosters and hens was at a high level (above 98%).

**Figure 1:**
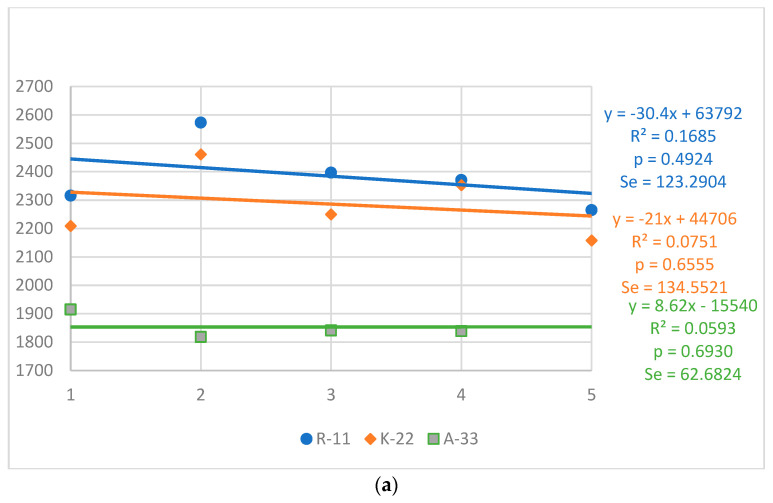

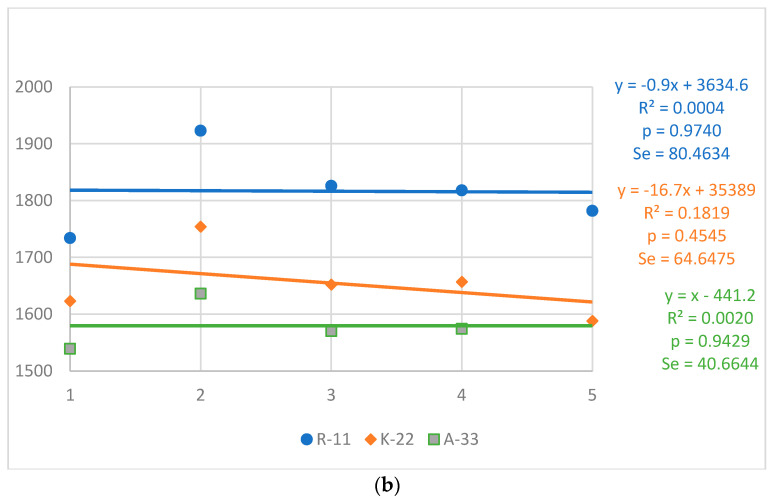
Strains of hens: Rhode Island Red (R-11 and K-22), Rhode Island White (A-33); Generations 1–5; R^2^—determination coefficient; *p*—significance of the trend line; S_e_—standard error of estimation. (**a**) Body weight at 20 weeks (g)—roosters; (**b**) Body weight at 20 weeks (g)—hens.

The trends in the body weight of roosters and hens by strain and year are shown in Figure 1a,b. The highest average body weight was shown by R-11 birds (2384 g for roosters and 1817 g for hens), and the lowest for A-33 roosters and hens, which were on average 512 and 238 g lower, respectively. During the studied time interval, in the K-22 and R-11 strains, the body weight of roosters showed a decreasing trend (21.0–30.4 g/year), while in the A-33 strain, it showed an increasing trend (8.62 g/year). In the R-11 and A-33 hen populations, body weight trends were stable, while the K-22 strain showed a clear negative trend (16.70 g/year). These model trends were not significant.

**Figure 2:**
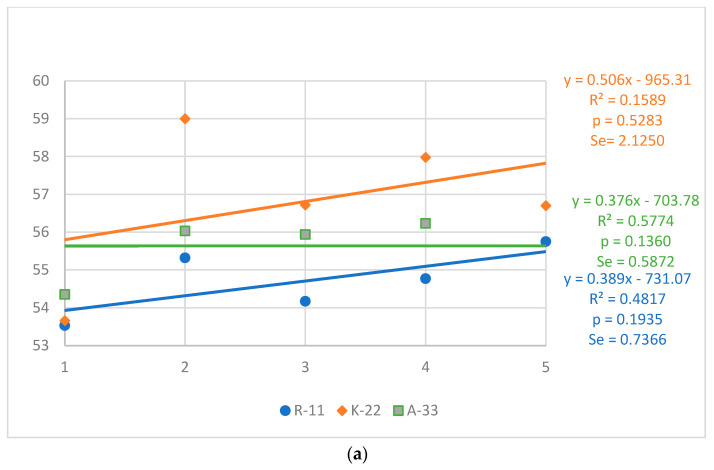

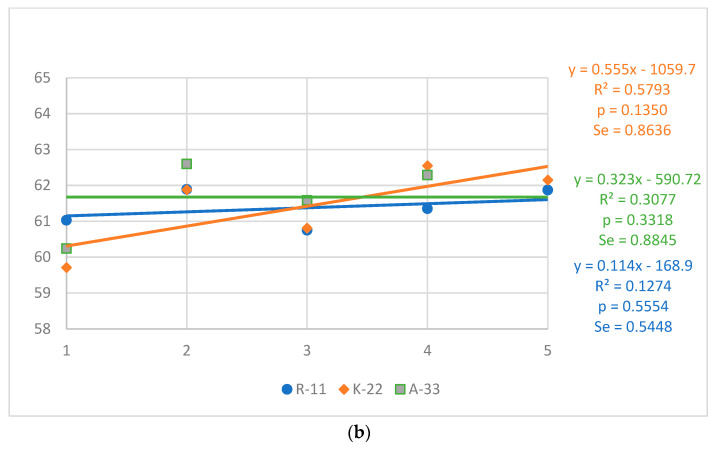
Strains of hens: Rhode Island Red (R-11 and K-22), Rhode Island White (A-33); Generations 1–5; R^2^—determination coefficient; *p*—significance of the trend line; S_e_—standard error of estimation. (**a**) Egg weight at 33 weeks (g); (**b**) Egg weight at 53 weeks (g).

The average egg weight (Figure 2a,b) assessed at 33 and 53 weeks ranged from 54.71 (R-11) to 56.81 g (K-22) and from 61.38 (R-11) to 61.74 g (A-33), respectively, with higher stability of the values at the second assessment date. Regardless of strain and evaluation date, the egg weight showed a positive trend. These model trends were not significant.

**Figure 3:**
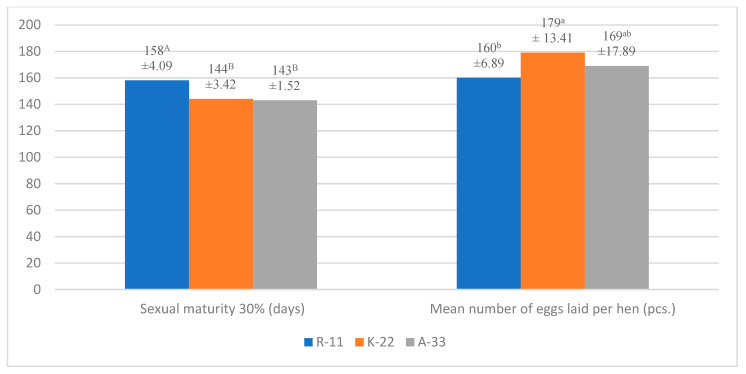
Sexual maturity at 30% laying (days) and mean number of eggs laid per hen over 36 weeks of production. Strains of hens: Rhode Island Red (R-11 and K-22), Rhode Island White (A-33); A, B—values for the same trait with different letters differ significantly (*p* ≤ 0.01); a, b—for *p* ≤ 0.05.

The sexual maturity, assessed as a 30% laying rate, and the mean number of eggs laid per hen is shown in Figure 3. The earliest egg production was observed in A-33 and K-22 hens (144 days on average, i.e., 20.6 weeks), and the latest in R-11 hens (158 days on average, i.e., 22.6 weeks). These changes had a direct impact on the number of laid eggs evaluated up to 56 weeks of age (36 weeks periods). The highest average laying rate was recorded in the A-33 (169 eggs/hen) and K-22 strains (179 eggs/hen), and the lowest in the R-11 strain (160 eggs/hen). The significance level was at *p* ≤ 0.05 between the breeds of hens R-11 and K-22.

**Figure 4:**
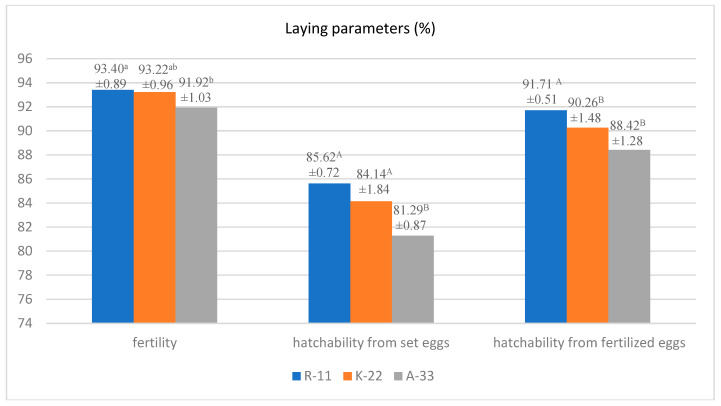
Strains of hens: Rhode Island Red (R-11, K-22), Rhode Island White (A-33); A, B—values for the same trait with different letters differ significantly (*p* ≤ 0.01); a, b—for *p* ≤ 0.05.

The hatchability parameters of the evaluated hen strains are shown in Figure 4. In the R-11 (93.40%) and K-22 (93.22%) hen populations, high rates of egg fertilization, a high hatchability of chicks from set eggs (85.62% and 84.14%), and high amounts of fertilized eggs (91.71% and 90.26%) were recorded. In contrast, in the A-33 strain, the evaluated parameters were lower at 91.92%, 81.29, and 88.42%, respectively.

The results of egg quality assessments performed at 33 and 53 weeks of the hens’ life are shown in Table 2 and Table 3. As the hens aged, there was a reduction in the shape index (by 0.32–3.88 percentage points). The average egg weight at 33 weeks ranged from 56.08 (R-11) to 58.70 g (K-22). The study showed genetically determined significant differences (*p* ≤ 0.01) between strains in eggshell color intensity (33.23–47.47%), with a tendency for the color to lighten with hens’ age (35.20–50.46%). The shell weight at 33 and 53 weeks ranged from 5.77 to 6.17 g and 6.47 to 6.80 g, respectively, with significant (*p* ≤ 0.01) statistical differences between evaluation dates. Significant reductions in eggshell thicknesses (318–346 vs. 269–291 μm) and densities (81.54–82.65 vs. 77.44–79.77 mg/cm^2^) were noted in all evaluated strains with the age of the hens (*p* ≤ 0.01). At 33 weeks, the shell strength ranged from 38.16 N (A-33) to 45.32 N (K-22). At the next evaluation date, the R-11 and K-22 strains showed a reduction in the shell strength from 3.80 to 4.22 N, respectively, and in the A-33 strain by 6.28 N (*p* ≤ 0.01). The increase in egg weight (from 6.23 to 9.27 g) was accompanied by a significant (*p* ≤ 0.01) increase in yolk weight (from 4.17 to 5.82 g). With the increase in age of the hens, all evaluated strains showed a significantly (*p* ≤ 0.01) higher yolk content (25.85–27.29 vs. 30.16–30.93%) and higher Roche yolk color scores (by 1.33–1.80 percentage points). At both the first and second evaluation dates, eggs from the tested hen strains were characterized by high albumen quality, evaluated by its height (9.04–9.31 vs. 8.08–8.34 mm) and Haugh units (94.48–95.84 vs. 88.13–88.55). The studies showed a significant (*p* ≤ 0.01) deterioration in the protein quality with the age of the hens. Only in eggs from R-11 hens evaluated at 53 weeks of age was there a higher proportion of blood spots (3.12%) and meat spots (6.25%).

## 4. Discussion

According to the “Genetic resources conservation program for laying hen populations”, endangered hen populations are protected by the in situ method, consisting of the conservation of live animals in their natural environment. In conservation flocks, no selection is carried out, so any changes occurring in the values of the studied traits are mainly due to the genetic characteristics of the breed and the environmental conditions in which the birds are housed. The main goal of the program for the conservation of laying hens’ genetic resources is to preserve individual populations of laying hens from extinction, by maintaining the genetic balance at an unchanging level in each protected flock (strain), while maintaining the characteristic phenotype of birds of both sexes [6,7]. A very important point in conducting breeding work in these flocks is the system of reproduction, based on the rotation of males between different groups of hens [16]. In our study, we obtained low inbreeding rates (Fx ≤ 0.17), proving that the rotational mating system used in the reproduction of four groups of birds effectively protects the evaluated hen populations from an increase in their inbreeding, consistent with our previous observations [16,25,26]. Spalona et al. [44] conducted a study on 41 local breeds of hens kept in Europe, and noted a much higher variation in the inbreeding rate (F_x_) than that presented in our work. Additionally, higher flock inbreeding rates (Fx = 0.7–1.9%) were shown by Pham et al. [12] for eight Taiwanese hen breeds. Vostrý et al. [14] emphasized that inbreeding is considered one of the production constraints due to its negative impact on the health and productivity of hens.

In our study, the health of the assessed breeds/strains, during both the rearing and production periods, was at a high level. It should be noted that in K-22 and A-33 hens, against the background of previous studies [16,26], the noted significant improvement in the health of flocks was the result of improving the environmental factors in which the birds were housed. These flocks were transferred in 2009 to a hen farm in Chorzelów, which has extensive experience in conservative breeding, which had a positive effect on improving both the health and production parameters. The data obtained testify to the good environmental conditions in which the birds were housed, appropriate food nutrition, and, most importantly, proper veterinary prophylaxis.

Body weight is the main trait of the breed, and its stability over a period of several years confirms the correct selection of birds for flock rotation [26]. Attention is drawn to the negative trends in body weight in K-22 roosters and R-11 hens; the development of this trait in subsequent generations should be observed. As indicated by Cywa-Benko [6] and Padhi [8], birds during the rearing period should grow rapidly to reach the optimal body weight for their type at the time of sexual maturity. Egg weight is generally positively correlated with the body weight of the birds, and the heritability rate (h^2^) has values of around 0.50 [45,46]. In our study, positive temporal trends in egg weight were observed in all evaluated strains, which is consistent with our previous observations [16]. The large variation in terms of cockerel and hen body and egg weights was indicated in the studies of Özdemir et al. [11], who conducted observations on six local hen breeds maintained in Italy and Turkey, and Padhi [8], who presented characteristics of selected native hen breeds maintained in Asia. Large differences in the age at which hens reached sexual maturity were found between the strains evaluated. The earliest egg production was reached by K-22 and A-33 hens, which also had the highest laying rate compared to R-11 hens, which in turn were distinguished by their higher body weight.

Over the course of five generations in all the evaluated strains, egg fertilization was at a high level, with also good hatchability of healthy chicks from set and fertilized eggs, which means a positive improvement in these indicators compared to our previous observations [25,26]. The results attest to the good health of the birds, a proper hatching technique, and good environmental conditions. As Szwaczkowski [47] points out, hatchability parameters are characterized by low heritability rates (h^2^ < 0.2). The stability of the reproductive traits of the native breeds of Polbar, Green-legged Partridge, Sussex, and Rhode Island Red was also indicated in the study of Gryzińska et al. [48]. Borzemska and Kosowska [49] reported hatching losses of 7.5 to 20%, depending on the species, breed, and bird use, which were assumed to be due to physiological causes.

The studies conducted indicate that there are significant differences in the physical characteristics of eggs from the hen strains evaluated. The significant increase in egg, yolk, and shell weight and size with the age of the hens is consistent with the results obtained by other authors [28,29,30,50]. In addition, one of the factors shaping egg weight is the rate of laying. A lower laying rate allows birds to store more material needed to build and increase egg weight [6,31]. The results of our own study also correspond with the results published by Nikolova and Kocevsky [51], who also noted that as hens age, there is a tendency for them to lay elongated eggs. According to Rizzi and Marangon [50], the weight of eggs from hens of native Italian breeds was 6–8 g less than eggs obtained from Hy-Line commercial hybrids.

In both the first and second evaluations, the eggs were characterized by very good albumen quality parameters. A significant deterioration in the albumen quality with the age of hens was indicated by Krawczyk and Calik [28] and Czaja and Gornowicz [30]. Biesiada-Drzazga and Janocha [33] state that the albumen quality in a fresh egg should be above 60 Haugh units; hence, it can be concluded that all eggs even from older hens obtained at 53 weeks of age were characterized by a high albumen quality. The intensity of yolk color is an important trait for the consumer, and it is mainly influenced by nutrition [34,35]. In our study, the hens were fed a mixture that was not additionally enriched with pigments, so the value of this trait was mainly influenced by genetic factors. Our study shows that eggs from older hens were characterized by more intense coloration, which is consistent with the results of Sokołowicz et al. [36]. In addition, older R-11 hens showed a higher frequency of blood and meat spots, which was also observed by Cywa-Benko [6].

Eggshell color is a trait correlated with the hen’s genotype, and its intensity is affected by age and the laying rate [28,40,41]. Studies have shown genetically determined differences in eggshell color intensity, with a tendency for the shell color to lighten with the age of the hens. The dark coloration of the shell is caused by the brown pigment ooporphyrin or protoporphyrin, which is derived from blood hemin, and the intensity of shell coloration is inversely proportional to the hens’ laying rate [29]. Additionally, Nedup and Phurba [52] report that the concentration of brown pigment decreases with longer laying periods. In addition, Sokołowicz et al. [36] observed that such a tendency applies more to eggs from native breeds of hens than to commercial hybrids, whose shell color is more stable. Commercially, the most important characteristics are the shell quality parameters, including the weight, thickness, and density of the shell, which affect its strength. In the literature, it has often been found that with the increase in age of the laying hens, the quality of eggshells significantly deteriorates, since an increase in the weight of the egg content is not accompanied by a corresponding increase in shell weight, resulting in a decrease in its strength [40,41]. This is also indicated by the results obtained, where a decrease was noted in shell quality parameters, including shell density, thickness, and strength, with the increase in age of the hens. As indicated by Drabik et al. [53], eggs with darker shells are characterized by a greater thickness and density. The existence of a positive relationship between shell strength and thickness and shell density is indicated by the results of Premavalli and Viswanagthan [54]. Biesiada-Drzazga et al. [55] observed a significant reduction in the shell thickness of eggs from Hy-Line Brown hens as the laying period progressed. A decrease in the eggshell strength with the age of the hens was also noted by Lewko et al. [56]. Hunton [40] and Lichniková and Zeman [57] showed that the decrease in eggshell quality with the age of hens is influenced by the decreased availability of calcium from feed in older hens, which results in a deterioration of the structure and concentration of matrix proteins and adversely affects the eggshell structure.

## 5. Conclusions

Based on these results, it was concluded that the evaluated hen populations (R-11, K-22, and A-33) are valuable strains, representing a reservoir of unique phenotypic and egg quality traits. It was shown that the random mating system used in the reproduction of flocks effectively protects the populations from an increase in the inbreeding rate. An influence of the hens’ origin (genotype) and age on the performance results, as well as egg quality traits, was found. Over five generations, the evaluated hen strains were characterized by high survival rates (above 98%). The study also found a large variation between strains R-11 and K-22 and strain A-33 in terms of the evaluated performance traits, especially body and egg weights, sexual maturity, and the laying performance of hens. The earliest to start laying were hens from the K-22 strains, who also showed a significantly (*p* ≤ 0.05) higher laying performance compared to R-11 hens. The results also indicate that the quality of eggs from hens of the compared strains varied. This was particularly true for such traits as shell color; egg, shell, and yolk weights; and shell quality. It was also shown that many egg and shell quality traits change with the age of the hens. The analysis of the obtained data indicates that the adopted methods of conservative breeding for these populations have led to the success of the conservation program.

## Figures and Tables

**Table 1:** The number of birds, the effective population size (N_e_), the inbreeding coefficient (F_x_), and the mortality and health-related deaths of the R-11, K-22, and A-33 hen populations during the rearing and production periods.

Strains	Generations	Number of Birds		N_e_	F_x_	Mortality and Health-Related Deaths (%)			
						Rearing Period		Production Period	
		♂	♀			♂	♀	♂	♀
R-11	1	84	720	300.90	0.17	1.00	2.38	0.00	0.69
	2	84	720	300.90	0.17	0.00	0.75	0.00	0.14
	3	100	800	355.56	0.14	1.00	1.75	0.00	1.11
	4	84	720	300.90	0.17	1.00	2.75	0.00	0.42
	5	84	720	300.90	0.17	3.00	2.75	0.00	1.25
	x	87.2	736	311.83	0.16	1.20	2.08	0.00	0.72
K-22	1	108	948	387.82	0.13	0.83	1.00	0.00	0.42
	2	108	948	387.82	0.13	0.00	0.82	0.93	0.21
	3	108	948	387.82	0.13	0.00	0.45	0.93	0.84
	4	108	948	387.82	0.13	1.67	2.18	0.00	0.84
	5	108	948	387.82	0.13	0.84	1.09	1.85	0.84
	x	108.0	948	387.82	0.13	0.67	1.11	0.74	0.63
A-33	1	96	864	345.60	0.14	0.00	3.80	1.04	0.93
	2	96	864	345.60	0.14	0.00	0.20	0.00	0.23
	3	96	864	345.60	0.14	0.83	1.60	1.04	1.50
	4	96	864	345.60	0.14	2.50	2.40	1.04	1.04
	5	96	864	345.60	0.14	0.00	2.20	0.00	1.16
	x	96.0	864	345.60	0.14	0.67	2.04	0.62	0.97

Strains of hens: Rhode Island Red (R-11 and K-22), Rhode Island White (A-33). x—long-term means for the strain.

**Table 2:** Shape index, egg weight, and egg shell quality.

Item	Breed (A)			Age (Weeks) (B)		A × B						SEM	Interactions		
	R-11	K-22	A-33	33	53	33			53				A	B	A × B
						R-11	K-22	A-33	R-11	K-22	A-33				
Shape index %	76.13 ^a^	75.03 ^b^	76.03 ^a^	76.72	74.74	78.07 ^A^	75.19 ^B^	76.92 ^B^	74.19 ^B^	74.87 ^A^	75.14 ^B^	0.218	0.045	<0.001	0.001
Egg weight g	60.35 ^Bb^	61.82 ^a^	62.34 ^A^	57.50	65.51	56.08 ^ab^	58.71 ^ab^	57.71 ^ab^	64.63 ^ab^	64.94 ^b^	66.98 ^a^	0.384	<0.01	<0.01	0.031
Shell color %	48.97 ^A^	36.83 ^B^	34.85 ^C^	38.40	42.03	47.47 ^B^	33.23 ^D^	34.50 ^D^	50.46 ^A^	40.43 ^C^	35.2 ^D^	0.568	<0.001	<0.001	<0.001
Shell weight g	6.12 ^Bb^	6.35 ^a^	6.46 ^A^	5.95	6.68	5.77	5.90	6.17	6.47	6.80	6.76	0.051	<0.01	<0.001	0.311
Shell thickness μm	318 ^A^	316 ^AB^	295 ^B^	336	284	346	344	318	291	289	269	0.003	<0.001	<0.001	0.693
Shell density mg/cm^2^	79.49	80.29	81.04	82.17	78.38	81.54	82.65	82.31	77.44	77.93	79.77	0.532	0.474	<0.001	0.676
Shell strength N	43.17 ^A^	43.21 ^A^	35.02 ^B^	42.85	38.08	45.06	45.32	38.16	41.26	41.10	31.88	0.765	<0.001	<0.001	0.741

Strains of hens: Rhode Island Red (R-11 and K-22), Rhode Island White (A-33); SEM—standard error of the mean; A, B, C, D—values in rows separately for breed (a), age (b), and A × B differ significantly (*p* ≤ 0.01); a, b—for *p* ≤ 0.05.

**Table 3:** Results of egg quality assessment.

Item	Breed (A)			Age (Weeks) (B)		A × B						SEM	Interactions		
	R-11	K-22	A-33	33	53	33			53				A	B	A × B
						R-11	K-22	A-33	R-11	K-22	A-33				
Yolk weight g	17.37	17.66	17.79	15.25	19.97	15.28 ^C^	15.57 ^C^	14.88 ^C^	19.45 ^B^	19.75 ^B^	20.70 ^A^	0.198	0.191	<0.001	<0.001
Yolk content %	28.73	28.47	28.39	26.56	30.49	27.29 ^B^	26.54 ^BC^	25.85 ^C^	30.16 ^A^	30.40 ^A^	30.93 ^A^	0.202	0.584	<0.001	<0.001
Yolk color pts	5.30 ^A^	4.67 ^B^	4.60 ^B^	4.03	5.68	4.40	4.00	3.70	6.20	5.33	5.50	0.082	<0.001	<0.001	0.104
Albumen height mm	8.56	8.72	8.88	9.19	8.24	9.04	9.24	9.31	8.08	8.19	8.44	0.117	0.522	<0.01	0.948
Haugh units jH	91.31	92.08	92.00	95.31	88.24	94.48	95.61	95.84	88.13	88.55	88.16	0.642	0.845	<0.001	0.902
Blood stains %	1.56	0.00	0.00	0.00	0.00	0.00	0.00	0.00	3.12	0.00	0.00	-	-	-	-
Meat stains %	3.12	0.00	0.00	0.00	2.08	0.00	0.00	0.00	6.25	0.00	0.00	-	-	-	-

Strains of hens: Rhode Island Red (R-11 and K-22), Rhode Island White (A-33); SEM—standard error of the means; A, B, C—values in rows separately for breed (a), age (b), and A × B differ significantly (*p* ≤ 0.01).

## Data Availability

Not applicable.
